# The context and practice of handwashing among new mothers in Serang, Indonesia: a formative research study

**DOI:** 10.1186/1471-2458-13-830

**Published:** 2013-09-11

**Authors:** Katie Greenland, Endang Iradati, Abigael Ati, Yanti Yulianti Maskoen, Robert Aunger

**Affiliations:** 1 Environmental Health Group, London School of Hygiene & Tropical Medicine, London, UK; 2 MCHIP-Maternal and Child Health Integrated Program, Jakarta, Indonesia; 3 Consultant for MCHIP- Maternal and Child Health Integrated Program (Jhpiego), Jakarta, Indonesia

**Keywords:** Hand-washing, Formative research, Behaviour change

## Abstract

**Background:**

This article reports on formative research into the context and practice of handwashing with soap by new mothers, which can substantially impact child morbidity and mortality. New mothers are an important target group for handwashing interventions: they are considered particularly susceptible to behaviour change and their actions can directly affect a child’s health.

**Methods:**

Twenty-seven mothers of infants (including neonates) from urban and rural sub-districts of Serang were recruited and filmed over a period of eight hours. Video footage was used to identify handwashing occasions and to understand the context in which behaviour took place. Each woman was subsequently interviewed.

**Results:**

Handwashing with soap was found to be infrequent, typically occurring after eating, cooking and household chores or after cleaning a child’s bottom. Handwashing before preparing food or eating was rare. Pre-pregnancy routines were reported to have been disrupted. Advice on child care comes from many sources, particularly the midwife and new child’s grandmother.

**Conclusions:**

Developing interventions to change perceptions and practice of handwashing would seed an important behaviour and could save lives. New mothers represent an ideal target group for such an intervention. We suggest that interventions target an increase in handwashing with soap after contact with own and a baby’s faecal matter as part of the post-defecation hygiene routines. As the child’s grandmother is an authoritative source of information about parenting, interventions focussed on improving newborn care could target grandmothers as well as midwives.

## Background

Handwashing with soap has been viewed as one of the most cost-effective ways of reducing the global infectious disease burden [1]. The benefits associated with handwashing with soap largely stem from reductions in diarrhoeal diseases [2-4], a common cause of morbidity and a leading cause of death among children under-five [5]. Despite the irrefutable evidence in favour of handwashing, it is rarely practiced at times when pathogen transfer could be interrupted, and even more rarely involves the use of soap [6].

Interventions that promote handwashing with soap are therefore vitally important to public health, yet changing behaviour is notoriously difficult. It may be possible to optimise the effectiveness of an intervention by concentrating efforts during a “teachable moment”, a concept from education describing a naturally-occurring event or life-stage that motivates a person to acquire new behaviours [7]. In 2002, McBride and colleagues examined the evidence for teachable moments using the example of smoking cessation [8]. They developed a model that characterised a teachable moment as an event which i) increases a person’s risk perception, ii) triggers a strong affective or emotional response, and iii) redefines their social role or self-concept. Disease diagnosis, hospitalisation and pregnancy were identified as teachable moments.

It is also well known in marketing circles that major changes in life-status such as getting married, divorced, having a child or reaching pensionable age are associated with significant changes in the pattern of consumption [9,10]. Markov chain analysis of large datasets available on household consumption in the USA suggests that the first major change in such patterns takes place when a household gains its first child [11].

As well as being a life-changing, emotionally-charged event, pregnancy is a time when women willingly adopt new behaviours to minimise health risks to both the mother and child; evidence suggests that other lifestyle change interventions can also be successfully implemented antenatally [12,13]. New motherhood could be viewed as a continuation of this teachable moment, particularly for primiparous women: they have an innate concern for the wellbeing of their baby and they desire to be - and to be viewed to be - a “good” mother.

As well as hypothesising that new and expectant mothers are particularly susceptible to behaviour change interventions, there are compelling public health reasons for focusing handwashing behaviour change interventions at this time in a woman’s life: 1) a mother’s hygiene behaviour directly impacts the health of her child, therefore early adoption of better hygiene practices that continue as the child grows could reduce morbidity and mortality from common diseases such as diarrhoea and respiratory infections [2,14-16]; 2) children learn important life-skills from their mother [17-19]; and 3) if intervention takes place early enough, it is plausible that handwashing could have an important impact on neonatal survival by reducing sepsis and tetanus [20]. A trial to quantify the impact of handwashing during the perinatal period on neonatal morbidity and mortality is currently underway (http://clinicaltrials.gov/ct2/show/NCT01309321?id=10036&rank=2) which should strengthen the evidence base for this third assertion.

Standard approaches to hygiene promotion - focused on educating people about germs and the value of using soap - have rarely resulted in positive, sustained behaviour change [2,6,21-23]. It is now increasingly recognised that hygiene behaviour is determined by a range of factors and is deeply-rooted in the environment in which the behaviour takes place [6]. Although the perinatal period could be an opportune moment to change behaviour, little is known about handwashing behaviour in this population with regards to how, when and where a new mother fits handwashing into her daily activities and what barriers to optimal practice exist (e.g. fatigue, disruptions of baby to daily routine etc.). To design effective hygiene promotion interventions targeting new and expectant mothers it is therefore important to carry out formative research [21,24-27]. The formative research described in this paper was conducted to gain insight into the daily lives and handwashing practices of new mothers in Indonesia, and to identify factors facilitating and hindering handwashing with soap in this population.

## Methods

### Study setting

Indonesia has more than 230 million inhabitants spread over 17,000 islands [28]. The formative research was conducted in Serang District, 72 km west of the capital Jakarta in Banten Province, Java, and an area where the local study partner (MCHIP – Maternal & Child Health Integrated Program) operates. Serang District includes the city of Serang and 28 sub-districts and has a total population of around 1.5 million. Two sub-districts (one urban and one rural) were randomly selected for the study: urban Kramatwatu and rural Pamarayan.

In Indonesia the infant mortality rate is 34 per 1000 live births. Infant mortality is higher in Banten Province than the national average, at 46 per 1000 live births [29]. Common childhood killers such as acute respiratory infections and diarrhoeal diseases are prevalent: 11% and 14% of children under five were reported to have experienced an episode of ARI or diarrhoea respectively in a two-week period [29]. In Indonesia, 88% of urban and 70% of rural households can access water on their premises, 21% and 5% of which (urban and rural respectively) have a piped water source. Data from the Indonesian Demographic and Health Survey indicates that 75% of urban households and 43% of rural households have a private toilet, although national figures mask great disparity between Provinces [29].

### Participant selection

Village midwife records from Community Health Centres in Kramatwatu and Pamarayan were used to identify a purposive sample of women who had given birth within the last year, including both primiparous and multiparous women, and infants as well as neonates. Potential participants were told the study aimed to learn how their life has changed since having children.

Informed consent was obtained from all participants and their husbands whenever possible. Ethical approval was granted by LSHTM Independent Ethics Committee. The Maternal and Child Directorate of the Ministry of Health Indonesia also supported and approved the study.

### Data collection

A range of tools can be employed for formative research. Due to our focus on how to introduce new hygiene behaviours into this teachable moment, we required good information about how new mothers currently engage in child-care and personal hygiene behaviours. This is very difficult to investigate through questioning. On the other hand, measuring behaviour accurately is also challenging; relying on self-report often produces implausible data [30,31], while structured observation is expensive and intrusive [32,33]. For these reasons, we opted to capture this information using direct observation (video recording).

#### Video recording

We used small hand-held video cameras (Panasonic SDR-S50) to directly observe behaviour. We piloted this method in India and Bangladesh and found it acceptable to participants and a valuable data source. Three local women were trained to carry out unobtrusive video recording. They were instructed to film the mother continuously during two periods: when she first woke (around 4 am) for five hours, and from 4 pm until after the evening meal (typically another three hours). Filming at these times maximised the opportunity to observe periods of intense activity in the household such as food preparation, hygiene routines and eating at least one meal. To ensure filming began when the respondent woke up, the field worker stayed in a respondent’s home the previous evening, something found to be culturally acceptable in this setting. Although this was the primary reason for arriving the night before, gaining familiarity with the respondent in this way may possibly reduce reactivity among mothers when filming actually began (it has been proposed that reactivity may be higher in the initial period of observation) [34].

Participants were not followed when they left the household compound, and any visitors to the home were not filmed unless they consented. Field workers were requested to keep the mother’s hands on camera whenever possible and to keep a record of any periods of absence from the compound to aid interpretation of video footage. Participants’ wishes for privacy were respected at all times, for example, by turning the camera away during breastfeeding and while in the bathroom.

#### Interviews

Semi-structured interviews were conducted the day after filming by four researchers (three female) working in two teams, each consisting of one LSHTM and one Indonesian researcher (the latter also acted as translators). Participants were questioned about their handwashing practices and motivations for handwashing, how their life has changed since having children, their knowledge of health risks and their sources of information about child-care.

If we want to insert or change a particular behaviour in a person’s routine, it is first important to understand the particular sequence of activities that take place currently – the “script”. In a process called “script elicitation” (used in cognitive psychology [35]), we asked primiparous women to describe their day from the time they wake in the morning to when they go to bed. Cards with simple drawings, made by the authors in advance, and depicting everyday activities and chores such as breastfeeding, doing laundry or cooking a meal, were laid out in front of the respondent as she talked, each card being laid down depicting the activity she had just mentioned. Once she had finished describing her day, the respondent was prompted about any obvious omissions (e.g. eating lunch) and the gaps in the routine were filled in. Inclusion of scripting framed discussion about *how* the young mothers’ daily lives had changed and suggested a variety of lines of questioning in interviews. The picture cards were also used as visual aids to help understand the flexibility of the daily routine, order of activities and to describe a typical day before having children.

### Data analysis

Videos were reviewed daily so that unclear portions of film could be immediately discussed and interpreted together with the field worker and with the participant (in interview the following day). A detailed sequence of events was recorded using an Excel database. The database was used to identify occasions when hands were washed with and without soap and to document details of the physical environment. Previous work in this field has shown us that hands may well be washed with soap, but the activities that prompt handwashing (and soap use) are not usually times considered important from a public health perspective. We decided to categorise handwashing behaviour according to whether or not soap was used to wash hands and whether or not hands were washed at key times so we could better describe handwashing behaviour. Each participant was categorised into one of five categories: Washer (washes hands with soap at least once at a critical time); Reactive Washer (washes hands with soap at least once but at a non-critical time, i.e. motivated by reasons such as dirt or smell on hands); Rinser (washes hands at least once at a critical time but using water only); Reactive Rinser (washes hands with water only, never at a critical time); and Avoider (never observed to handwash). These categories best described their hand-washing behaviour based on the times when they washed their hands and whether or not they were ever seen to use soap to hand-wash on this occasion. Categorising individuals in this way allows the data to be viewed differently, illustrating the range of behaviours that take place and the differences between individuals in terms of whether and when soap is used to wash hands.

We performed thematic analysis of interview transcripts to provide insights into practices and underlying motives and drivers of behaviour. This analysis allowed unexpected themes and concepts to emerge from the information provided by informants. Scripts (daily routines) from the different women were aggregated to find the most frequent sequence of daily events, thus generating a “master” routine that illustrates the course of life before and after giving birth to a first child. All aspects of this study conform to the RATS guidelines for qualitative research.

## Results

### Characteristics of participants

Twenty-seven new mothers were filmed and interviewed, 15 in urban Kramatwatu and 12 in rural Pamarayan. Only two families were approached and refused to participate. Participants were aged between 18 and 39, all were literate and all had completed at least primary education, while two were university graduates. Monthly household income varied widely, from < US$115 to US$920. The mean monthly household income (collected in categories and converted from Indonesian Rupiah) was US$230-345 in urban households and < $115 in rural households, comparable with the general population. Half of the participants (n = 14) were first time mothers, eight of whom had returned to their maternal homes for an extended perinatal period as is customary in this region. The median age of the infant being cared for was two months (range 13 days to nine months); eleven were neonates (Table 1).

**Table 1:** Overview of the characteristics and handwashing practices of new mothers, Serang, Indonesia (n = 27)

**HW status**	**Age**	**Setting (urban/rural)**	**Education**	**Monthly income (USD)**	**Other household residents**	**No. of children**	**Age of children (range)**	**Main HW facility**	**Occasions when handwashing was observed *at least once *(W = only water used, S = soap used)**
Washer	38	Urban	n/a	$345-$460	none	3	n/a	kitchen sink	after returning home (S); after sweeping (S); *food preparation (S); after eating (S) *after baby defecation (bathes baby) (S)
	37	Urban	high school	$690-$805	none	3	15y/2 m	kitchen sink	after taking out rubbish (S); after laundry (W); *food preparation (S); after eating (S); *after baby defecation (S, also uses wet wipes);*before breastfeeding (S?); after returning home (W)
	21	Urban	high school	$115-$230	father, brother	1	1.5 m	bathroom	after cleaning bathroom floor (S); after sweeping/taking out rubbish (S); *before eating (S); after eating (S?); before breastfeeding (wet wipes)
	22	Urban	high school	$345-$460	none	1	2 m	tap (corner of kitchen)	*before handling baby (stops cooking to respond to baby) (S); after eating (S); after rinsing out baby bath (before preparing baby bottle) (W); *before eating(W); other unknown reasons (W)
	26	Urban	high school	$115-$230	none	2	3.5y/21d	kitchen sink	after laundry (S); *before eating (S); after dishes (W); *after baby defecation (S); *before breastfeeding (W)
	33	Urban	academic	$460-$575	none	3	13y/2 m	kitchen sink	*before eating (S); *before serving (W)
	38	Urban	high school	$115-$230	none	4	15y/2 m	kitchen sink	after dishes (S); *before breastfeeding (S)
	39	Rural	university	$920	none	4	12y/13d	HW stand bedroom & store in kitchen (carries ladle to wash in sink)	after returning home *(then eating) (S); after eating (W); *after baby defecation (S); *before breastfeeding (S)
	25	Rural	high school	$230-$345	parents, brother	1	3 m	kitchen sink	after laundry (S); *before eating (S); after eating (S); *after baby defecation (S); *before handling baby (after cooking) (W)
Reactive washer	22	Urban	high school	$230-$345	in-laws	1	4 m	bathroom	after cooking (chili) (S); after eating (S)
	29	Urban	junior high	$115-$230	none	1	9 m	bathroom	after cleaning floor (S); after eating (W)
	32	Urban	academic	$460-$575	none	2	7y/17d	kitchen (no sink)	after sweeping (S); after dusting (W); after laundry & hanging out clothes (W); after wiping table (W); after eating (S?)
	35	Urban	university	$230-$345	none	3	7y/2 m	kitchen sink	*after baby defecation (W); after returning home (S)
	25	Rural	high school	<$115	none	2	5y/28d	store in kitchen	after eating (S)
	18	Rural	junior high	<$115	parents	1	28d	store in kitchen	after eating (S); after washing dishes (W)
Rinser	20	Rural	elementary	$115-$230	parents, brother, sister	1	28d	store in kitchen	after eating (W); *after defecating (W - at well, open defecator)
	18	Rural	junior high	<$115	none	1	28d	bathroom	after moving laundry (W); *(during) food prep (W); after clearing fallen food (W only? too dark to see); after dishes (W); *before serving (W)
Reactive rinser	32	Urban	high school	$230-$345	yes (unknown)	2	8y/2 m	kitchen sink	after sweeping (W); after laundry (W)
	18	Rural	elementary	<$115	mother	1	3 m	store in kitchen	after sweeping (W); after feeding baby puree (W); after eating (W)
Avoider	35	Urban	high school	$115-$230	mother	2	8y/21d	kitchen sink	no handwashing observed
	23	Urban	junior high	$115-$230	none	1	3 m	kitchen sink	no handwashing observed
	34	Urban	elementary	$115-$230	none	3	13y/14d	bathroom	no handwashing observed
	18	Rural	elementary	$345-$460	parents, sister	1	2 m	store in kitchen	after eating (W - right hand only)
	23	Rural	high school	<$115	parents	2	7y/45d	store in kitchen	after clearing dishes (W)
	19	Rural	elementary	<$115	mother-in-law	1	2 m	store in kitchen	after eating (W)
	23	Rural	high school	<$115	parents, brother	1	28d	store in kitchen?	after eating (W?)
	25	Rural	elementary	<$115	grandparents, brother	1	28d	store in kitchen	after serving (W - right hand only)

A "washer" washes hands with soap (at least once) on at least one hand-wash occasion (e.g., after baby defecation, before eating); a "reactive washer" washes hands with soap (at least once) at a non-essential time (e.g., after sweeping); a "rinser" washes hands without soap (at least once) on at least one hand-wash occasion (e.g., after baby defecation, before eating); a "reactive rinser" washes hands without soap (at least once) at a non-essential time (e.g. after sweeping); and an "avoider" does not wash hands at all or only rinses hands after eating.

An asterisk is used to indicate handwashing occasions of public health importance. For the purpose of this study, breastfeeding and handling the baby are considered to be important handwashing occasions.

Note: the 35-year-old woman classified as a dirt washer could also have been classified as a rinser (handwash without soap after baby defecation).

### Handwashing practice

Each individual was assigned to one of five categories based on characteristics of their hand-washing practice (Table 1). Although these categories are largely illustrative, this scheme divides up the observed variation in practice in ways likely to be connected to the determinants of practice.

The eight to nine hours of video footage from each participant revealed that hands were washed at least once by all but three women. Half the participants (15 of 27) used soap on at least one occasion. Overall, nine participants were classified as “washers”, six were “reactive washers”, two were “rinsers”, two were “reactive rinsers” and eight were “avoiders” (Table 1). Handwashing often required a change of room, most frequently taking place in the kitchen (12 participants used a tap/sink, 10 used stored water), although five participants used a tap or stored water in the bathroom. Handwashing – with and without soap – took place in response to a variety of different events: handling food, defecation (own and the baby), household activities, returning home from outside, and breastfeeding (table). Handwashing was observed more frequently and on more occasions in urban households than in rural households (the majority of washers were urban). In rural households, with one exception, hands were washed only after eating or cleaning. Handwashing and soap use do not appear linked to a child’s age (neonate or other infant) or number of children in a household (Table 1). Interviews revealed that soap is not perceived to be expensive and film showed special baby soap or other products were used when bathing the baby; wet wipes were also used by some mothers. The use of such products indicates that this population is more affluent than you would find in some other low-income settings.

#### Food-related events

Food was almost always prepared early in the morning and stored in a cupboard or under netting until it was eaten, when it was served into bowls and eaten with a spoon; rice was occasionally served using bare hands and was sometimes eaten by hand. Hands were rarely washed before food preparation, serving others, or before eating, but sixteen participants washed hands with water or soap after eating, making it the most common time that handwashing took place (see Table 2), transcending the handwashing categories (Table 1). In interview, women explained that they rinse hands before eating to stop rice from sticking, and after cooking and eating to remove bad smells. Mothers also expressed concern that hands that have contacted chilli pepper can “make the baby hot” and should be washed with soap, a behaviour that was also observed on video.

**Table 2:** Frequency of handwashing with water only and handwashing with water and soap among new mothers, by type of handwashing occasion (N = 27)

**Event**	**No. events observed**	**No. (%) events accompanied by handwashing with water only**	**No. participants handwashing with water only**	**No. (%) events accompanied by handwashing with soap**	**No. participants handwashing with soap**
**Food-related**					
Before food preparation	38	0	0	2 (5%)	2
Before serving food	51	2 (4%)	2	0	0
Before eating	52	2 (4%)	1	5 (10%)	4
After cooking	34	2 (6%)	1	3 (9%)	2
After eating	39	8 (21%)	7	9 (23%)	9
Defecation-related					
After cleaning baby's bottom/dirty napkin	28	7 (25%)	1	8 (32%)	5*
After own defecation	1	1 (100%)	1	0	0
Housework/environment-related					
After cleaning (e.g. sweeping, taking out rubbish)	54	4 (7%)	4	10 (19%)	5
After washing dishes	21	5 (24%)	3	2 (7%)	1
After doing laundry	23	8 (30%)	4	2 (9%)	2
After returning home	11	1 (9%)	1	3 (27%)	3
Other					
Before breastfeeding	148	1 (0.7%)	1	2 (1.4%)	2*

*wet wipes also used by one woman. Cleaning included sweeping, putting out rubbish, and the use of chemical cleaning products.

The proportion of events accompanied by handwashing (with and without soap) were calculated from behaviours directly observed on video footage (approx. eight hours per participant). The end of an evening meal was not always captured on film at the request of the participant or due to the need to relocate to the next household. For this reason after eating events were observed less frequently than before eating events.

Participants did not consistently wash hands or use soap in response to any event.

#### Housework-related events

Soap was also observed being used after completing housework or doing laundry, and on three occasions after returning home from errands in urban households. Women rationalised this by explaining that adult clothing is dirty and must be kept separate from the baby’s laundry (also observed on film), and that they come into contact with dirty things or people when they go into town.

#### Defecation-related events

In almost all households the latrine and handwashing station were in the bathroom so it was not possible to observe whether hands were washed post-defecation. On one occasion a mother who defecated in a defecation pond – a small pond with a hanging latrine behind the house - was observed to rinse her hands with water upon returning to the house (Table 2).

Handwashing following cleaning a child’s bottom after defecation was seen on film following 15 of 28 observed defecation events (Table 2). Babies’ nappies were changed in a variety of locations but often in the same way. The five women who used soap or a wet wipe were classified as “washers”. They typically dealt with the event immediately, scrubbing the faeces from the cloth napkin in the bathroom or placing the napkin in a pile of laundry and then washing hands, often before bathing the baby, a time-consuming process of cleaning the child. One woman (a university graduate) had a handwashing station in the bedroom near where she changed the baby’s clothing. She placed the soiled/wet napkins in a basket underneath this handwashing station and washed hands afterwards, occasionally using soap. This woman was inconsistent in her handwashing practice and use of soap, but it appears that the presence of the handwashing station cued her behaviour because not all women washed hands at this time, and she herself did not wash hands on any other occasion with such regularity.

#### Other events

Handwashing before “handling the baby” was reportedly advised by the midwife, although mothers’ admitted to forgetting or not having time to wash hands then, particularly when the baby is crying; three women washed hands or used a wet wipe before breastfeeding (Table 2), a practice also reported to have been advised by local midwives.

### Life-style changes

**Figure 1:**
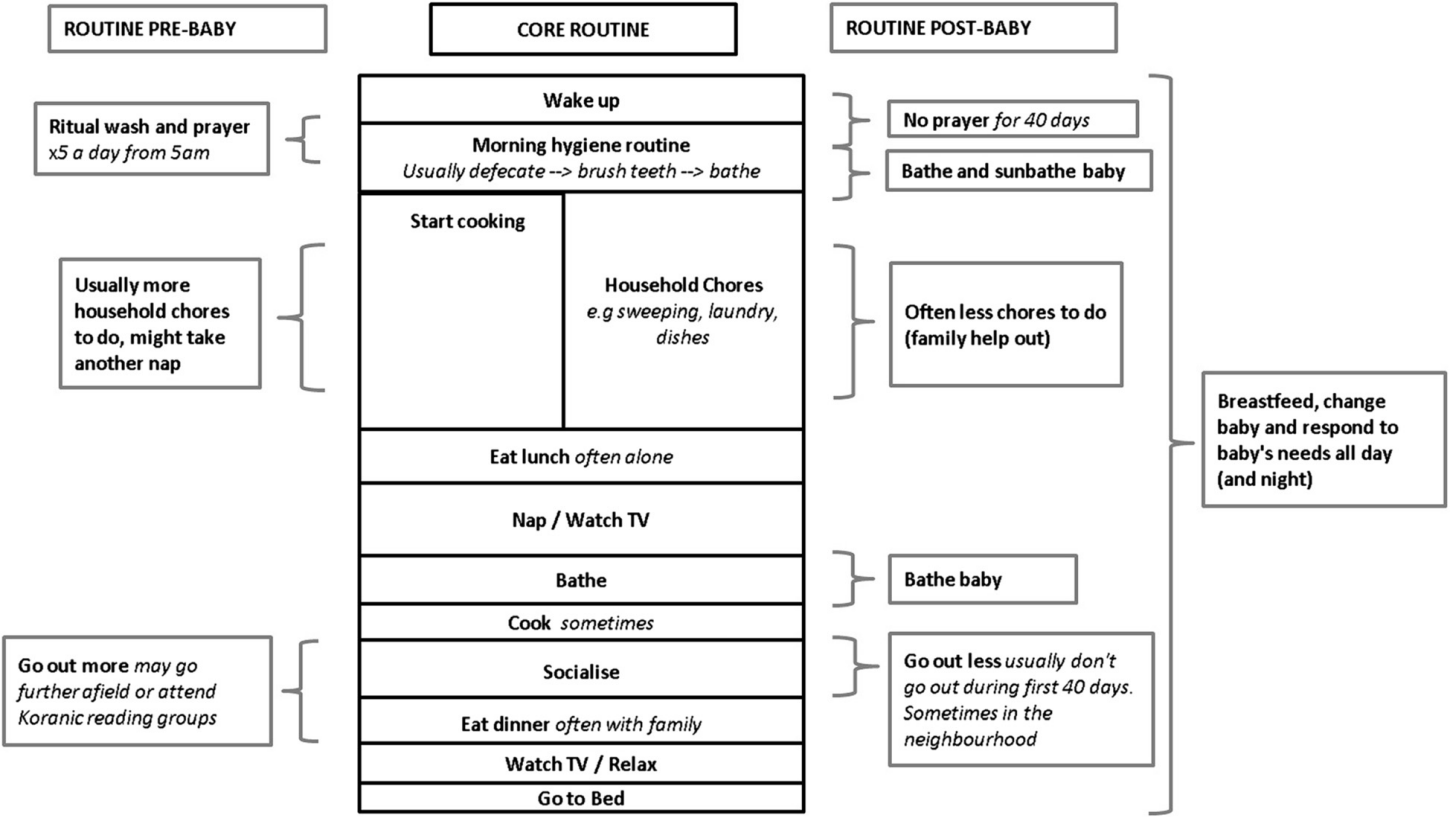
Schematic to show typical activities in the daily routine of a woman before and after she has a baby.

In this section we consider how a new baby has impacted the life of the mother and the influence of her social world on her behaviour in order to provide important context for a handwashing intervention targeting this population. Figure 1 shows the typical daily routine for first-time mothers before and after giving birth according to the scripts. Women reported that they lack sleep, no longer go out (particularly during the first 40 days postpartum when they are supposed to remain indoors according to Islamic beliefs and local custom) and generally spend their time caring for their child; they particularly reported enjoying the twice-daily bathing of the baby, an activity that is diligently performed. In general, routines are very regular and similar across all participants; the main difference being the wake-up time (around 4 am to almost 6 am) and the subsequent timing of daily activities. Mealtimes and household chores are slotted in around the baby’s needs, particularly breakfast and lunch, which are typically eaten alone.

During interview women reported deliberately resting more and changing their diet during pregnancy (eating more vegetables and less spicy food) for the sake of their own health and that of their offspring. Mothers with neonates were observed on film to do very little other than care for their new charge, explaining in interview that they are supposed to spend extended periods of time sitting during the postpartum period to promote healing, and that household chores such as ironing and laundry are consequently taken over by other family members. Video footage and family structure (Table 1) support these assertions, although multiparous mothers of neonates were generally more active than their primiparous counterparts. Interviews and video footage revealed the strong influence the mother, mother-in-law and midwife have on a new mother’s actions. Advice is offered on numerous topics, including guidance on how best to recuperate (to sit upright with legs out), how to burp the baby, to sunbathe the baby each morning (to prevent jaundice), to drink *Jamu* – traditional herbal remedy sold on the street – and to avoid eating fruit. The majority of mothers reported obediently following these recommendations even though they often could not explain their purpose. Several multiparous mothers recalled how much more concerned they were to follow advice when they gave birth to their first child than they are now that they know what they are doing. The most valued advice reportedly comes from the midwife who is viewed as a reputable source of information. Midwives instruct on how to bathe the baby (they are concerned about “spraining” the baby when they handle it), preventing cord infection and the importance of breastfeeding. The instructions they give on preventing cord infection and breastfeeding mention handwashing. Immediately post-birth in some rural areas a traditional birth attendant may provide services such as washing the mother’s clothing and helping to care for and bathe the child. Women reported receiving conflicting advice from different sources, and despite the value placed on information given by midwives, family members, particularly a woman’s mother or mother-in-law are also important influences. Video footage showed grandmothers play an active role in preparing food and feeding young infants, and were frequently heard issuing instructions in the background on how to bathe the baby and clean up faeces.

Women have a wide range of beliefs concerning the health risks their child faces. They were particularly concerned about the child becoming ‘sprained’ when they are handled due to the child’s ‘floppy neck’, but also fear fever, diarrhoea and infection from other children or from the cord. When asked explicitly what they could do to prevent illness, particularly diarrhoea, women felt there was little they could do and that childhood illness is related to the child passing through different developmental stages. Hygiene was rarely mentioned. Further questioning on whether handwashing could prevent illness was met with confusion or a “no”.

## Discussion

This formative research was designed to capture detailed information on the lives and handwashing practices of new mothers in Serang, Indonesia. Handwashing with soap occurs at a low level, but is not constrained by water or soap availability. Hands are typically washed after eating, cooking, doing household chores and after cleaning a child: times when hands are visibly dirty, oily, smelly, sticky or otherwise uncomfortable. We divided the participants into handwashing ‘types’ (such as those who use soap only when they are motivated by hands which are visibly dirty or smell – so-called ‘reactive washers’) to illustrate the range of hygiene behaviours that take place with respect to hands, and the differences between individuals in terms of whether and when soap is used to wash hands. We believe this categorization is insightful and may be true of other populations, and so could usefully guide program development on handwashing behaviour in future projects. This classification may also have implications for health promotion efforts, as it is possible that each of these types will require different kinds of intervention, since they appear to be motivated to use soap in different kinds of circumstances. While it is difficult to make specific recommendations for other handwashing programs without knowing their objectives, we believe that any ability to predict the types of handwashers likely to be found in a population can assist other programs, especially if they are not able to conduct research themselves. Knowing such types has program implications – for example, reactive washers are likely to be motivated by disgust at visible contamination on their hands, while avoiders seem not to respond to such cues to handwash, and so will require other means to change their behaviour.

Emotional drivers of behaviour are also important determinants of handwashing [36]. Observation of when hands are washed, supported by interview responses about why hands are washed, lead us to hypothesise that handwashing at these times is most likely to be driven by feelings of “disgust” directed at substances or smells perceived to have contaminated hands; and “comfort”, desire for hands to feel clean [6]. “Disgust” is therefore also the probable motivator for urban women who wash hands after returning home, ridding hands of dirt from the environment or other people they have contacted, while rinsing hands after doing laundry could be to remove the harsh feeling of detergent (“comfort”). Conversely, washing hands that look and feel clean derives little benefit, which fits with the low levels of handwashing observed at relevant food-hygiene junctures (i.e. before cooking or eating). Observed handwashing behaviour is consistent with other low-income countries [6,37] and other studies in Indonesia [38].

Both the video footage and interviews indicate that handwashing is infrequent and does not seem to be prompted by having a new child, although “nurture” (desire to care for one’s offspring) has been previously demonstrated to drive maternal handwashing behaviour [6]. Failure to wash hands is not because of a lack of time; these mothers have considerable time on their hands, being almost exclusively concerned with child-care, and having been relieved of other responsibilities by others in the household. Rather, hand washing may not be seen as a necessary part of being a good mother in this society. Sporadic instances of handwashing/using baby wipes before breastfeeding are likely to be an attempt to practice a behaviour desired by the midwife: information provided in interviews about the advice midwives gave concerning handwashing before breastfeeding thus matched the behaviours observed on film. The same mother was observed to respond differently to different child defecation events, possibly due to whether or not hands were contaminated with faeces. In a previous study in Burkina Faso, the stools of young children were regarded as less offensive than the stools of older children [39]. It is possible that mothers do not find their infant’s faeces disgusting and for this reason “disgust” may not be a strong driver of handwashing behaviour at this time. It would have been interesting to have collected more information on this. However, encouraging the mother to handwash after clearing up a baby’s stool would hopefully translate into correct hygiene behaviour later in life. Although hands are allegedly washed after cooking with chilli for the protection of the child, it is more probable that handwashing after cooking is habitual.

The automaticity of existing handwashing behaviour [36] is one reason why it is hard to change. A “habit” can be defined as a behaviour that is performed frequently in a constant context [40]. The context winds up cueing the behaviour so it occurs automatically in that situation. This means that habits are context-dependent. When the context changes existing habits are disrupted, providing an opportune time to insert new behaviours and form new habits [41]. Our findings confirm that new motherhood results in many changes to a woman’s daily routine and diet, giving us reason to be optimistic about the potential for behaviour change at this time. They also confirm that new motherhood is an appropriate teachable moment as defined by McBride [8]: women are aware of and concerned about health risks their child faces; their social role has changed; and they adore their new baby, undoubtedly a strong emotional response. New mothers are likely to prove a receptive audience if they can be convinced of the benefit of an intervention: although handwashing behaviour at present does not appear to have changed as a result of the new baby, “nurture” motives could be stimulated if women see handwashing as a trait of a “good mother” or they believe it will be beneficial to their child’s health. This could be an important campaign angle. Primiparous women are particularly open to new advice and would be a relevant target. Furthermore, as women willingly follow the advice of health professionals and family members, it could be relevant for midwives – who frequently contact women during the peri-natal period – to be involved in delivery of a community-based intervention to improve hand hygiene in this population, and for that intervention to also target influential family members. The frequency of contact with the health system at this time could be useful for reinforcing handwashing messages.

Further, rather than attempt to introduce handwashing with soap before breastfeeding (one of the current recommendation from midwives), which happens too frequently to be constantly interrupted by trips to a handwashing location, it is contact with faeces that should be the primary concern if the desire is to set a handwashing habit that will benefit the child’s health at a later date. In addition, we know from the videos that considerable time and care is already invested in cleaning and dressing babies in some households after a defecation event. The videos also showed us how variable handwashing practices were, even within the same individual, partially due to the presence of cues such as the physical setting, presence of an object, or visually dirty hands. Baskets for the various clothes and ointments needed at this time are a part of every new mother’s ‘kit’ for newborn childcare in Indonesia. It would probably be relatively easy to insert handwashing with soap soon after cleaning the baby’s bottom and before extensive further contact with the child. This is likely to be especially true if there is a handwash stand or other visual reminder present in a relevant location within the household as well. As we observed these cleaning and changing rituals being explicitly taught to young mothers by their mothers, it is natural to target these mothers-of-mothers as the appropriate channel for communicating the need to include handwashing with soap as part of normal child-care operations.

This study had some obvious limitations. After every interview a discussion took place to clarify any points of confusion. However, we cannot exclude the possibility that the presence of “foreigners” during interviews may have influenced respondents, nor the fact that bias can also stem from local researchers’ perceptions of their own culture. The collection of video footage provided a rich data source, although the challenge of filming at first light and the inability to capture activity occurring inside a closed bathroom remained drawbacks, the latter a perennial difficulty in studies of handwashing behaviour [34,42]. Although some degree of reactivity might be expected in any study of behaviour [34,42], it was only evident in three films where the women in question were clearly performing tasks they had not done previously in an attempt to look good. In one particular film, her mother could be heard issuing instructions in the background. It is difficult to know what other activities women did or did not do because they were being filmed, but we did not see obvious posing for the camera, women performed multiple activities many times in the same way on film, and they did not know exactly what behaviour we were interested in capturing. As participants were identified using the village midwife’s records, we have not included women who do not access antenatal services or those who give birth at home without a skilled birth attendant. As accessing antenatal services and choosing to be helped by a skilled attendant are strongly correlated with income, education, and living in an urban or rural locality [43], and handwashing practices in this study and other studies are associated with the same factors, it is possible that we would have seen different behaviour if these women had been included.

## Conclusions

This study used formative research methods to provide information on the lives and hygiene practices of new mothers, an important, yet under-exploited target group for handwashing interventions. Current handwashing rates are low, but handwashing facilities are available, and the ubiquity of women’s willingness to change other behaviours at this time bodes well for the possibility of incorporating handwashing with soap into their daily routines. If an intervention is developed it will also be important to decide when in the peri-natal period it should be delivered, as women are inundated with messages during pregnancy and the early stages of motherhood. We recommend efforts to promote handwashing focus on introducing handwashing into the newly-formed routine around baby defecation events, involving the grandmother(s) to provide support and authority.

## Competing interests

The authors declare that they have no competing interests.

## Authors’ contributions

RA designed the study with contributions from EI. KG performed analysis and drafted the manuscript. All authors were involved in data collection and read and approved drafts of the manuscript, including the final version.

## Pre-publication history

The pre-publication history for this paper can be accessed here:

http://www.biomedcentral.com/1471-2458/13/830/prepub
